# Sequence-based mutation patterns at 41 Y chromosomal STRs in 2 548 father–son pairs

**DOI:** 10.1093/fsr/owad016

**Published:** 2023-05-31

**Authors:** Ze Liu, Guannan Long, Yubo Lang, Dahua Liu, Biao Zhang, Shaobo Yu, Fei Guo

**Affiliations:** DNA Laboratory of Forensic Science Center, Shenyang Public Security Bureau, Shenyang, China; DNA Laboratory of Forensic Science Center, Shenyang Public Security Bureau, Shenyang, China; School of Public Security Information Technology and Intelligence, Criminal Investigation Police University of China, Shenyang, China; Department of Forensic Medicine, Jinzhou Medical University, Jinzhou, China; DNA Laboratory of Forensic Science Center, Shenyang Public Security Bureau, Shenyang, China; DNA Laboratory of Forensic Science Center, Shenyang Public Security Bureau, Shenyang, China; School of Forensic Science and Technology, Criminal Investigation Police University of China, Shenyang, China

**Keywords:** Y chromosomal short tandem repeat (Y-STR), mutation, Northern Han Chinese, Goldeneye™ DNA ID Y Plus Kit, ForenSeq™ DNA Signature Prep Kit, StatsY software

## Abstract

A total of 2 548 unrelated healthy father–son pairs from a Northern Han Chinese population were genotyped at 41 Y chromosomal short tandem repeat (Y-STRs) including DYS19, DYS388, DYS389I, DYS389II, DYS390, DYS391, DYS392, DYS393, DYS437, DYS438, DYS439, DYS444, DYS447, DYS448, DYS449, DYS456, DYS458, DYS460, DYS481, DYS518, DYS522, DYS549, DYS533, DYS557, DYS570, DYS576, DYS593, DYS596, DYS627, DYS635, DYS643, DYS645, Y-GATA-H4, DYF387S1a/b, DYF404S1a/b, DYS385a/b, and DYS527a/b. In 2 548 father samples, 2 387 unique haplotypes were detected with the haplotype diversity and discrimination capacity values of 0.999 956 608 and 0.96 741 007. The average gene diversity (GD) value was 0.6934 with a range from 0.1051 at DYS645 to 0.9657 at DYS385a/b. When comparing alleles at 24 overlapped Y-STRs between the ForenSeq™ deoxyribonucleic acid (DNA) Signature Prep Kit on the MiSeq FGx® Forensic Genomics System and the Goldeneye® DNA ID Y Plus Kit on the Applied Biosystems™ 3730 DNA Analyzer from 308 father samples in mutational pairs, 258 alleles were detected by massively parallel sequencing (MPS) typing including 156 length-based alleles that could be obtained by capillary electrophoresis (CE) typing, 95 repeat region (RR) variant alleles and seven flanking region variant alleles. Hereof, we found 16 novel RR variant alleles and firstly identified two SNPs (rs2016239814 at DYS19 and rs2089968964 at DYS448) and one 4-bp deletion (rs2053269960 at DYS439) that had been validated by the Database of Short Genetic Variation. Sanger sequencing or MPS was employed to confirm 356 mutations from 104 468 allele transfers generated from CE, where 96.63% resulted in one-step mutations, 2.25% in two-step, and 1.12% in multi-step, and the overall ratio of repeat gains *versus* losses was balanced (173 gains *vs.* 183 losses). In 308 father–son pairs, 268 pairs occurred mutations at a single locus, 33 pairs at two loci, six pairs at three loci, and one pair at four loci. The average Y-STR mutation rate at 41 Y-STRs was ⁓3.4 × 10^−3^ (95% confidence intervals: 3.1 × 10^−3^–3.8 × 10^−3^). The mutation rates at DYS576 and DYS627 were higher than 1 × 10^−2^ in Northern Han Chinese, whilst the mutation rates at DYF387S1a/b, DYF404S1a/b, DYS449, DYS518, and DYS570 were lower than initially defined. In this study, the classical molecular factors (the longer STR region, the more complex motif and the order father) were confirmed to drive Y-STR mutation rates increased, but the length of repeat unit did not conform to the convention. Lastly, the interactive graphical and installable StatsY was developed to facilitate forensic scientists to automatically calculate allele and haplotype frequencies, forensic parameters, and mutation rates at Y-STRs.

**Key points:**

- 308 of 2 548 father–son pairs from Northern Han Chinese occurred at least one mutation(s) across 41 Y-STRs.
- Sanger sequencing or MPS was employed to confirm those mutations generated from CE.
- The longer STR region, the more complex motif and the order father drove Y-STR mutation rates increased.
- StatsY was developed to calculate allele and haplotype frequencies, forensic parameters and mutation rates at Y-STRs.

## Introduction

The Y chromosomal short tandem repeat (Y-STR) markers are wildly used in forensic casework and kinship analyses due to their non-recombination nature and paternal transmission pattern [1]. Normally, males carry single Y-chromosomes (ChrY), which are passed down from their fathers and transmitted to their sons without changing as haplotypes if the haplotypes are located in non-recombining region of ChrY and have no mutational events. Analyses of Y-STRs are beneficial in examining of a mixture with male minor component and female major component in sexual assault cases, determining the number of male suspects in “gang rape” investigations, and widening dragnets for male question samples (“Q”) without suspects (“K”) cases, missing persons cases, and mass disaster victim identifications by familial searching [2].

Since Roewer et al. [3] reported the first Y-STR locus in 1992, thousands of Y STR markers have been identified across the ChrY [4]. A core set termed as the “minimal haplotype” loci was selected in 1997, including DYS19, DYS389I, DYS389II, DYS390, DYS391, DYS392, DYS393, and DYS385a/b [5, 6]. In 2003, the Scientific Working Group on DNA Analysis Methods (SWGDAM) recommended a core set “extended haplotype” that included the “minimal haplotype” loci plus DYS438 and DYS439 [2]. On the basis of these recommendations, a series of commercial kits incorporating 12–38 Y-STR loci have been released by Promega [7, 8] and Thermo Fisher Scientific [9–11]. In China, Peoplespot, AGCU, HEALTH Gene Technologies, and Microread have sequentially developed their commercial Y-STR kits including 36–41 loci [12–15]. Although the number of loci seems more enough, a match between a question sample and a suspect with Y-STRs does not carry the same resolution of differentiation as a match with autosomal STRs would, because Y-STRs only identify a patrilineage rather than an individual. In 2012, Ballantyne et al. [16] identified a new panel, namely the rapidly mutating (RM) Y-STRs with higher mutation rates above 1 × 10^−2^, which are expected to increase the resolution of patrilineage differentiation compared to conventional Y-STR markers. Besides length-based deoxyribonucleic acid (DNA) typing assays, massively parallel sequencing (MPS) analysis has potential to provide more genetic information *via* repeat and flanking region sequences. Zhao et al. [17] and Kwon et al. [18] have successfully detected sequence-based Y-STRs using in-house panels on Ion Torrent Personal Genome Machine (PGM) and MiSeq System, respectively. In 2015, Verogen (former Illumina) launched the ForenSeq™ DNA Signature Prep Kit where 26 Y-STRs can be sequenced along with ˃200 forensically relevant markers [19, 20]. The novel marker (i.e. RM Y-STR) and cutting-edge technology (i.e. MPS) would facilitate to expand the ChrY application in forensic community.

In this study, the Goldeneye® DNA ID Y Plus Kit was employed to create a set of Northern Han Chinese haplotypes and estimate mutation rates from a large number of father–son pairs. It can co-amplify 41 Y-STR loci, including 11 SWGDAM loci, 21 commonly used loci (DYS388, DYS437, DYS444, DYS447, DYS448, DYS456, DYS458, DYS460, DYS481, DYS522, DYS527a/b, DYS533, DYS549, DYS557, DYS593, DYS596, DYS635, DYS643, DYS645, and Y-GATA-H4) and nine RM Y-STR loci (DYF387S1a/b, DYF404S1a/b, DYS449, DYS518, DYS570, DYS576, and DYS627). Also, Sanger sequencing or MPS was employed to confirm those mutations generated from capillary electrophoresis (CE). At the same time, we compiled the software, namely StatsY, to facilitate the workflow for analysis of population statistics and mutation rate estimations at Y-STRs.

## Materials and methods

### Population

Liaoning is a province of the People's Republic of China, located in the northeast of the country (38°N–43°N; 118°E–125°E) with the Yellow Sea in the south, North Korea in the southeast, Jilin to the northeast, Hebei to the southwest, and Inner Mongolia to the northwest. The population of Liaoning is mostly Northern Han Chinese (36 169 617, 84.92%) with minorities of Manchu (5 385 287, 11.94%), Mongolian (669 972, 1.59%), Hui (264 407, 0.59%), Korean (241 052, 0.54%), and Xibe (132 615, 0.30%) at the 2020 census [21]. Bloodstain samples of 2 548 unrelated healthy father–son pairs were collected from Liaoning Province after informed consent and approval of the Ethical Committee of Jinzhou Medical University (No. 202001-6). All male germ-line transmissions were confirmed with a minimum paternity index of 10 000 using the Goldeneye® DNA ID 20A Kit (Peoplespot, Beijing, China) according to the manufacturer’s recommendations [22].

### CE-Y-STR typing

Samples were prepared using 1.2 mm discs punched from bloodstains on Whatman® FTA® cards (GE, Piscataway, NJ, USA) and were directly amplified without DNA extraction by Goldeneye® DNA ID Y Plus Kit (Peoplespot) in 10 μL of reaction volume containing 2 μL of 5 × polymerase chain reaction (PCR) Reaction Mix, 2 μL of 5 × Y Plus Primer Mix, and 6 μL ddH_2_O. Thermal cycling was performed on the GeneAmp™ System 9700 (Thermo Fisher Scientific, Foster City, CA, USA) using the following conditions: denaturation for 2 min at 95°C, amplification for 30 cycles of 5 s at 94°C, 45 s at 60°C and 45 s at 72°C, extension for 15 min at 60°C and hold at 15°C. One microliter of PCR products or allelic ladders were diluted in 8.5 μL Hi-Di™ Formamide mixed with 0.5 μL ORG500 Size Standard. After denaturation for 3 min at 95°C and quenching on ice for 3 min, all prepared samples were separated and detected on the Applied Biosystems™ 3730 DNA Analyzer (Thermo Fisher Scientific). Standard run parameters involved: dye set at J6, sample injection for 8 s at 1.2 kV and electrophoresis in the 36 cm capillary using POP-7™ polymer as indicated in the GeneMapper36_POP7 run module. CE raw data were analyzed using GeneMapper™ *ID-X* Software v1.5 (Thermo Fisher Scientific) with peak amplitude threshold set at 50 relative fluorescent units.

### MPS-Y-STR typing

Bloodstain samples from mutational father–son pairs were extracted using the magnetic beads on the Microlab STAR Liquid Handling System (Hamilton, Bonaduz, Switzerland). Genomic DNA was quantified on the Applied Biosystems™ QuantStudio™ 5 Real-Time PCR System (Thermo Fisher Scientific) using the Quantifiler® Trio DNA Quantification Kit (Thermo Fisher Scientific) according to the manufacturer’s recommendations [23]. Library preparation was performed using the ForenSeq™ DNA Signature Prep Kit (Verogen, San Diego, CA, USA) according to the manufacturer’s recommendations [24]. Hereof, one nanogram purified DNA (5 μL input volume at a concentration of 0.2 ng/μL) was amplified using DNA Primer Mix A. MPS was performed on the MiSeq FGx® Forensic Genomics System (Verogen) using the MiSeq FGx® Reagent Kit following the manufacturer’s instruction [25] with two modifications: (i) 52 libraries were pooled for a run, including 50 samples, a positive control, and a negative control; (ii) 7 μL pooled libraries was added into 591 μL Hybridization Buffer (HT1) and then mixed with 4 μL Human Sequencing Control (HSC) mixture, not 2 μL HSC in the previous study [20]. MPS raw data were processed by the ForenSeq™ Universal Analysis Software (UAS) v1.3 (Verogen) at default analysis thresholds [26].

### Sanger sequencing

Samples were amplified using primers as listed in Supplementary Table S1. Amplicons were directly sequenced after purification or cloned using the TOPO® TA Cloning® Kit (Thermo Fisher Scientific) according to the manufacturer’s recommendations. Sanger sequencing was performed on the Applied Biosystems® 3130*xl* Genetic Analyzer (Thermo Fisher Scientific) using the BigDye® Terminator v3.1 Cycle Sequencing Kit (Thermo Fisher Scientific) following the manufacturer’s instructions. Raw data were analyzed using the Sequencing Analysis Software v5.3.1 (Thermo Fisher Scientific).

### Statistical analysis

Allele and haplotype frequencies were estimated and shared haplotypes were screened from father samples by mere counting. The multi-copy markers (DYF387S1a/b, DYF404S1a/b, DYS385a/b, and DYS527a/b) were treated as allelic combinations. Fraction of unique haplotype (F_UH_) was calculated as: F_UH_ = *N_unique_*/*N_diff_* where *N_unique_* is the number of unique haplotypes and *N_diff_* is the number of different haplotypes. Haplotype diversity (HD) or gene diversity (GD) was determined as: HD or GD = [*n/*(*n–*1)] (1−*∑p_i_^2^*) where *n* is the population size and *p_i_* is the frequency of the *i*th haplotype or allele [27]. Discrimination capacity (DC) was determined as: DC = *N_diff_*/*n* where *N_diff_* is the number of different haplotypes and *n* is the population size.

The number of mutations, the number of one/two/multi-step mutation(s) and the number of gains/losses were screened by mere counting. Mutation rates were calculated as the number of mutations between father–son pairs divided by the number of allele transmissions per each Y-STR marker. Confidence intervals (CI) of the mutation rates were estimated using the exact binomial probability distribution. Herein, the allele of DYS389II and the allele of DYS389II subtracted by the value of DYS389I were calculated, respectively; the number of allele transmissions for the multi-copy markers (DYF387S1a/b, DYF404S1a/b, DYS385a/b, and DYS527a/b) was counted as twice that of meioses.

All of allele and haplotype frequencies, forensic parameters, and mutation rates were automatically calculated using StatsY v1.0. It is freely available at https://www.researchgate.net/publication/359440373_StatsY_v10.

Chi-square test, Fisher’s exact test, two-sample *t*-test, and linear regression were computed using R software version 4.0.5 [28] and figures were generated by Package “ggplot2” and “vcd” for R.

## Results and discussion

### Haplotypes

#### Allele frequencies and GD values

Allele frequencies and GD values at each locus from 2 548 father samples are listed in Supplementary Table S2A. A total of 358 alleles were observed at 41 Y-STRs and allele frequencies ranged from 0.0002 to 0.9447 (allele 8 at DYS645). The number of alleles at each locus ranged from 5 at DYS593 and DYS645 to 18 at DYS385a/b. Six intermediate alleles were observed: allele 19.2 at DYS448 and allele 30.2 at DYS449 had been documented on the Y Chromosome Haplotype Reference Database (YHRD) [29] already; alleles 12.2, 13.2, and 15.2 at DYF404S1a/b and allele 23.2 at DYS527a/b are not included in the YHRD and not reported in other literatures at the current time. Supplementary Table S2B showed details of allelic combinations at multi-copy loci, where DYS385a/b was considered as the most informative locus (85 allelic combinations) with 18 separated alleles and DYF404S1a/b was the lowest (37 allelic combinations) with 11 separated alleles.

The average GD value was 0.6934 with the range from 0.1051 at DYS645 to 0.9657 at DYS385a/b. The highest GD values were observed at the single-copy locus DYS449 (GD = 0.8853) and at the multi-copy locus DYS385a/b (GD = 0.9657), respectively. The lower GD values (GD < 0.5) were observed at four single-copy loci: DYS391 (GD = 0.3657), DYS437 (GD = 0.4989), DYS438 (GD = 0.4349) and DYS645 (GD = 0.1051), which were also reported in Southern Han Chinese [30] and Southeastern Han Chinese [31]. Moreover, all RM Y-STRs (GD > 0.7) and all multi-copy Y-STRs (GD > 0.9) exhibited high polymorphisms in Northern Han Chinese in this study.

#### Haplotype frequencies and forensic parameters

Totally, 2 465 different haplotypes were found amongst 2 548 father samples in Supplementary Table S3, where 2 387 haplotypes (96.84%) were unique, 73 (2.96%) were shared by two individuals and five (0.20%) by three. Five haplotypes (H00001–H00005) were shared by three individuals who had the same surnames but 19 autosomal STRs genotyping excluded these three individuals as being close relatives. Amongst 73 shared haplotypes (H00006–H00078), 61 haplotypes were shared by two individuals who had the same surnames but were also excluded as close relatives and 12 shared haplotypes (H00007, H00010, H00015, H00019, H00020, H00039, H00048, HT00061, H00062, H00066, H00070, and H00074) came from different surnames. Haplotype frequencies ranged from 0.0004 (unique haplotypes) to 0.0012 (shared haplotypes by three individuals).

The HD value was approximately equal to 0.999 956 608 and DC was 0.9674. Table 1 summarized statistics of shared haplotypes and forensic parameters amongst seven combined Y-STR systems. Generally, more unique haplotypes and higher forensic parameters were observed with the number of Y-STRs increased. Compared with AmpFℓSTR Yfiler Kit, N_diff_, F_UH_, HD, and DC increased by 10.00%, 7.65%, 0.01%, and 10.98%, respectively. The amplitudes of increasing were mild or very slow when comparing with 25–29 Y-STRs. These improvements in haplotype resolution and forensic parameters are similar to those from Southeastern Han Chinese [31] and Shandong Han Chinese [32].

**Table 1:** Haplotype distributions and forensic parameters for different combined Y-STR systems (*n* = 2 548).

Shared Haplotype	Combined Y-STR system						
	MH	SWGDAM	AmpFℓSTR Yfiler	PowerPlex Y23 System	AmpF**ℓ**STR Yfiler Plus	YHRD Ymax	Goldeneye DNA ID Y Plus
1	1 257	1 544	1 998	2 176	2 323	2 328	2 387
2	241	226	167	131	91	90	73
3	72	67	31	19	13	12	5
4	34	21	14	10	1	1	
5	18	20	7	1			
6	12	3	1				
7	7	2	1				
8	5	4		1			
9	3	1	1				
10	1	3	1				
11	1						
13	1	1					
14	1	1					
15		1					
18	1						
19	2						
21	1						
22		1					
24	1						
30	1						
N_L_	9	11	17	25	27	29	41
N_diff_	1 659	1 895	2 241	2 338	2 428	2 431	2 465
F_UH_	0.7 577	0.8 148	0.8 996	0.9 307	0.9 568	0.9 576	0.9 684
HD	0.999 021 341	0.999 477 129	0.999 816 843	0.999 893 853	0.999 941 242	0.999 942 522	0.999 956 608
DC	0.651 1	0.743 7	0.871 7	0.917 6	0.952 9	0.954 1	0.967 4

MH: minimal haplotype; SWGDAM: Scientific Working Group on DNA Analysis Methods; YHRD Ymax: a set of markers represents all available markers at Y Chromosome Haplotype Reference Database; N_L_: number of loci in the combined system; N_diff_: number of different haplotypes; HD: haplotype diversity; F_UH_: fraction of unique haplotype; DC: discrimination capacity; Y-STR: Y chromosomal short tandem repeat.

#### MPS typing versus CE typing

**Figure 1:**
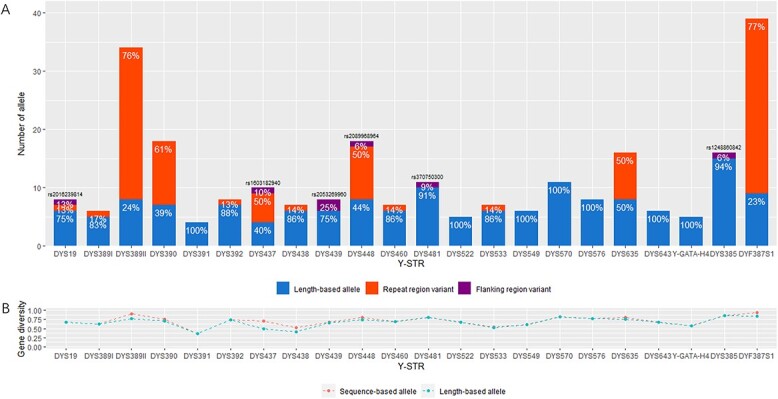
Comparison of 24 overlapped Y chromosomal short tandem repeat between the ForenSeq™ deoxyribonucleic acid (DNA) Signature Prep Kit and the Goldeneye® DNA ID Y Plus Kit from 308 father samples in mutational pairs. (A) Number of sequence-based alleles *versus* length-based alleles at each locus. Herein, sequence-based alleles by massively parallel sequencing typing are composed of length-based alleles that can be generated from capillary electrophoresis typing, repeat region variant alleles and flanking region variant alleles. (B) Gene diversity of sequence-based alleles *versus* length-based alleles at each locus.

In this study, 13 of 24 Y-STRs exhibited RR variants and contributed to 36.82% polymorphisms in total from 10 compound-complex repeat motif markers (DYS19, DYS389I, DYS389II, DYS390, DYS392, DYS437, DYS448, DYS635, and DYF387S1a/b) and three simple repeat motif markers (DYS438, DYS460, and DYS533). Figure 1 illustrated that the most RR variant alleles (30 alleles) were observed at DYF387S1a/b and the second most (26 alleles) at DYS389II. By comparing observed repeat motifs with reported ones from the STRbase (https://strbase.nist.gov/) and previous studies [17, 18, 33–40], Table 2 listed that 16 novel RR variant alleles were found at nine Y-STRs (DYS389II, DYS390, DYS392, DYS437, DYS448, DYS460, DYS533, DYS635, and DYF387S1a/b).

**Table 2:** Novel repeat region variant alleles found in this study (*N* = 308 from father samples).

Locus	Allele	Motif	Count	Frequency
DYS389II	28	[TAGA]11 [CAGA]2 N48 [TAGA]11 [CAGA]4	1	0.0032
	33	[TAGA]11 [CAGA]3 N48 [TAGA]15 [CAGA]4	1	0.0032
DYS390	21	[TAGA]13 [CAGA]8	1	0.0032
DYS392	14	[ATA]13 AAA	1	0.0032
DYS437	14	[TCTA]8 TCTG CCTG [TCTA]4	1	0.0032
	15	[TCTA]8 [TCTG]3 [TCTA]4	1	0.0032
DYS448	19.2	[AGAGAT]11 N38 [AGAGAT]9^a^	1	0.0032
	20	TGAGAT [AGAGAT]11 N42 [AGAGAT]8	2	0.0065
	23	[AGAGAT]14 N42 [AGAGAT]9	1	0.0032
DYS460	10	[CTAT]9 GTAT CTAT	1	0.0032
DYS533	11	TATC TATA [TATC]9	1	0.0032
DYS635	21	[TAGA]9 [TACA]4 [TAGA]2 [TACA]2 [TAGA]4	1	0.0032
	23	[TAGA]11 [TACA]4 [TAGA]2 [TACA]2 [TAGA]4	5	0.0162
	24	[TAGA]12 [TACA]4 [TAGA]2 [TACA]2 [TAGA]4	2	0.0065
DYF387S1a/b	38	[AAAG]3 GTAG [GAAG]4 [AAAG]2 GAAG [AAAG]2 [GAAG]7 [AAAG]18	1	0.0016
	40	[AAAG]3 GTAG [GAAG]4 [AAAG]2 GAAG AAAG [GAAG]11 [AAAG]17	1	0.0016

a N38: [atagag]2 [agatag]3 ataga- —gaa; Human genome assembly coordinate: GRCh38 Y:22219024-22219027 [TAGA/−] deletion.

As shown in Figure 1 and Table 3, six Y-STRs presented FR variants including four SNPs at DYS19, DYS437, DYS448, and DYS481 as well as two deletions at DYS439 and DYS385a/b, which provided 2.71% polymorphisms as a whole. Two SNPs (rs2016239814 at DYS19 and rs2089968964 at DYS448) and one 4-bp deletion (rs2053269960 at DYS439) were newly found in this study and have been validated by the Database of Short Genetic Variation (dbSNP) and then released in Build 155. One SNP (rs1603182940 at DYS437) and one 4-bp deletion (rs1248860842 at DYS385b) were firstly submitted to the dbSNP from a Korean population (ss3943499107) and a Swedish population (ss3020959847), respectively, but both were firstly reported in a Han Chinese population in this study.

**Table 3:** Flanking region (FR) variant alleles observed in this study (*N* = 308 from father samples).

Locus	GRCh38 coordinate [Ref/Alt]	FR variant	Count	Frequency	FR sequence reported by UAS 1.3
DYS19	Y:9684338 [G/ 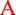 ]	**rs2016239814** ^**a**^ (ss4035869495)	1	0.0032	TGGTCAATCTCTGCACCTGGAAATAGTGGCTGGGGCACCAGGAGTAATACTTCGGGCCATGGCCATGTAGT 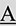 AGGACAAGGAGTCCATCTGGGTTAAGGAGAGTGTCACTATA **[TCTA]12 ccta [TCTA]3**
DYS437	Y:12346421 [G/ 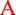 ]	rs1603182940 (ss4035869496)	2	0.0065	ATGCCCATCCGG **[TCTA]8 [TCTG]2 [TCTA]4** TCATCTATCATCTGTGAATGATGTCTATCTACTTATCTATGAATGATATTTATCTGTGGTTATCTATCTATCTATATCATCTGTGAATGACAGG 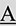 TCTTCCTCTG
DYS439	Y:12403513–12 403 516 [AGAA/ 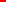 ] deletion	**rs2053269960** ^**a**^ (ss4035869497)	2	0.0130	GATAGATATACAGATAGATAGATACATAGGTGGAGACAGATAGATGATAAAT---- **[GATA]12** GAAAGTATAAGTAAAGAGATGAT
			2		GATAGATATACAGATAGATAGATACATAGGTGGAGACAGATAGATGATAAAT---- **[GATA]11** GAAAGTATAAGTAAAGAGATGAT
DYS448	Y:22218908 [T/ 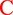 ]	**rs2089968964** ^**a**^ (ss4035869503)	1	0.0032	GAGA 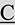 AGAGACATGGATAA **[AGAGAT]10 N42 [AGAGAT]8** AGAGAGGTAAAGATAGAGATAAATTTCCAG
DYS481	Y:8558336 [G/ 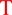 ]	rs370750300	2	0.0065	TGGCTAACGCTGTTCAGCATGCT 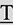 **[CTT]23** TTTTGA
DYS385a/b	Y:18639757–18 639 760 [CCTT/ 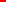 ] deletion	rs1248860842 (ss4035869499)	1	0.0016	TCCTTTCTTTTTCTC **[TTTC]11** ----CCTTCCTTCCTTCCTTCCTTTCTTTCTCTTTCCTCTTTCTCTTTCTTCTCTTTCTTTCTTTTTCTCTTTTTCTCTTTCTTTCTTTTTTACTTTCTTTCTCCTTCCTTCCTTCCTTTCTGAATTTCATTTCTTTTCTTT

The bold sequences represent repeat regions; the underlined bases in grey represent the mutations; the hyphens in sequences represent deletions.

a Newly found SNPs in this study have been validated by the dbSNP and released in Build 155.

Although RR variants and FR variants existed, alleles with these variants can be accurately and backward-compatibly called by the ForenSeq UAS. Further, MPS typing contributed to increasing the average GD value (0.7071) compared with that (0.6733) obtained by CE typing as shown in Supplementary Table S4. DYS437 showed the largest increment (42.19%) amongst the GD values and was also reported by Kwon et al. [18]. DYF387S1a/b achieved the highest GD value by sequences. However, other forensic parameters by sequences (F_UH_ = 1.0000, HD = 1.0000 and DC = 1.0000) remained the same as those by lengths because all of 308 haplotypes were unique with two methods.

### Mutations

#### Mutation rates

The mutation rates at 41 Y-STR loci were shown in Table 4. A total of 104 468 allele transfers in 2 548 father–son pairs were observed with 356 sequence-confirmed mutations (Supplementary Table S5). The average mutation rate was 3.4 × 10^−3^ (Binomial 95% CIs: 3.1 × 10^−3^–3.8 × 10^−3^). The estimated mutation rates ranged from 0.0 at DYS392 and DYS596 to 12.6 × 10^−3^ at DYS627 in Northern Han Chinese. The most mutable markers were observed at two RM Y-STRs (DYS576 and DYS627), which were similar to previous studies in Han Chinese populations [30, 31, 41–43]. Although DYF387S1a/b (1.59 × 10^−2^), DYF404S1a/b (1.25 × 10^−2^), DYS449 (1.22 × 10^−2^), DYS518 (1.84 × 10^−2^) and DYS570 (1.24 × 10^−2^) were initially identified as RM Y-STRs (μ > 1 × 10^−2^) by Ballantyne et al. [16], mutation rates were not as high as expected in Northern Han Chinese. Hereof, DYS449 (9.0 × 10^−3^), DYS518 (9.0 × 10^−3^) and DYS570 (5.1 × 10^−3^) presented as fast mutating Y-STRs (μ: 5 × 10^−3^–1 × 10^−2^), which were also reported in Southeastern and Henan Han Chinese populations [31, 44]; DYF387S1a/b (4.9 × 10^−3^) and DYF404S1a/b (3.3 × 10^−3^) presented as moderately mutating Y-STRs (μ: 1 × 10^−3^–5 × 10^−3^) and a relatively lower mutation rate at DYF387S1a/b was reported as well in Southern, Southeastern, Hunan, Henan, and Beijing Han Chinese populations [30, 31, 42, 44, 45]. Thus, mutation rates at initially defined RM Y-STRs may vary amongst different populations.

**Table 4:** Estimated mutation rates at 41 Y chromosomal short tandem repeat in Northern Han Chinese.

Y-STR loci	Number of allele transfers	Number of mutations	Number of one-step mutations	Number of two-step mutations	Number of multi-step mutations	Number of gains	Number of losses	Mutations rates (×10^−3^)	Binomial 95% CIs (×10^−3^)
DYS19	2 548	7	7	0	0	5	2	2.7	1.1–5.7
DYS388	2 548	9	9	0	0	3	6	3.5	1.6–6.7
DYS389I	2 548	7	7	0	0	6	1	2.7	1.1–5.7
DYS389II	2 548	20	19	1	0	10	10	7.8	4.8–12.1
DYS390	2 548	6	6	0	0	2	4	2.4	0.9–5.1
DYS391	2 548	4	4	0	0	2	2	1.6	0.4–4.0
DYS392	2 548	0	0	0	0	0	0	0.0	0–1.4.0
DYS393	2 548	3	3	0	0	1	2	1.2	0.2–3.4
DYS437	2 548	2	2	0	0	0	2	0.8	0.1–2.8
DYS438	2 548	2	2	0	0	2	0	0.8	0.1–2.8
DYS439	2 548	4	4	0	0	1	3	1.6	0.4–4.0
DYS444	2 548	4	4	0	0	1	3	1.6	0.4–4.0
DYS447	2 548	6	5	1	0	1	5	2.4	0.9–5.1
DYS448	2 548	2	2	0	0	1	1	0.8	0.1–2.8
DYS449	2 548	23	23	0	0	18	5	9.0	5.7–13.5
DYS456	2 548	9	9	0	0	4	5	3.5	1.6–6.7
DYS458	2 548	11	10	1	0	6	5	4.3	2.2–7.7
DYS460	2 548	9	9	0	0	3	6	3.5	1.6–6.7
DYS481	2 548	5	5	0	0	3	2	2.0	0.6–4.6
DYS518	2 548	23	22	0	1	10	13	9.0	5.7–13.5
DYS522	2 548	4	4	0	0	1	3	1.6	0.4–4.0
DYS549	2 548	2	2	0	0	0	2	0.8	0.1–2.8
DYS533	2 548	8	8	0	0	3	5	3.1	1.4–6.2
DYS557	2 548	8	8	0	0	5	3	3.1	1.4–6.2
DYS570	2 548	13	12	1	0	6	7	5.1	2.7–8.7
DYS576	2 548	27	27	0	0	10	17	10.6	7.0–15.4
DYS593	2 548	3	3	0	0	2	1	1.2	0.2–3.4
DYS596	2 548	0	0	0	0	0	0	0.0	0.0–1.4
DYS627	2 548	32	31	1	0	14	18	12.6	8.6–17.7
DYS635	2 548	11	11	0	0	5	6	4.3	2.2–7.7
DYS643	2 548	3	3	0	0	1	2	1.2	0.2–3.4
DYS645	2 548	5	5	0	0	1	4	2.0	0.6–4.6
Y-GATA-H4	2 548	8	8	0	0	4	4	3.1	1.4–6.2
DYF387S1a/b	5 096	25	23	0	2	15	10	4.9	3.2–7.2
DYF404S1a/b	5 096	17	16	1	0	6	11	3.3	1.9–5.3
DYS385a/b	5 096	13	12	1	0	9	4	2.6	1.4–4.4
DYS527a/b	5 096	21	19	1	1	12	9	4.1	2.6–6.3
Total	104 468	356	344	8	4	173	183	3.4	3.1–3.8

A total of 356 mutational events were observed in 308 father–son pairs, where 268 pairs occurred mutations at a single locus, 33 pairs at two loci, six pairs at three loci and one pair at four loci. It indicated that 12.09% (308/2 548) father–son pairs could be discriminated using 41 Y-STRs. Also, a total of 375 stepwise mutations were counted (Table 4), amongst which 344 mutations (96.63%) belonged to one-step mutations, eight (2.25%) to two-step, two (0.56%) to three-step, and each one (0.28%) of four-step and five-step. The percentage of one-step mutations at 41 Y-STRs was similar to that at 27 Y-STRs (95.20%) from Southern Han Chinese [30], that at 42 Y-STRs (97.72%) from Eastern Han Chinese [41], that at 50 Y-STRs (97.80%) from Hunan Han Chinese [42] and that at 62 Y-STRs (97.29%) from Southeastern Han Chinese [31], and there was no statistically significant difference between the percentage of one-step and multi-step mutations amongst those Han Chinese populations (*P* = 0.8104, Fisher’s exact test). The result fitted to the stepwise mutation model. The overall ratio of repeat gains *versus* losses was approximately balanced, with a ratio of 1:1.06 (173 gains *vs.* 183 losses in Table 4), which is consistent with previous studies [30, 31, 44].

#### Mutation patterns

Mutation patterns were investigated the correlation between mutation rates and molecular factors as below: (i) length of STR region (i.e. allele repeat number); (ii) complexity of repeat motif (i.e. simple, compound, and complex repeats); (iii) length of repeat unit; (iv) age of father at the gametogenesis (i.e. the birth year of the father subtracted from that of the son).

**Figure 2:**
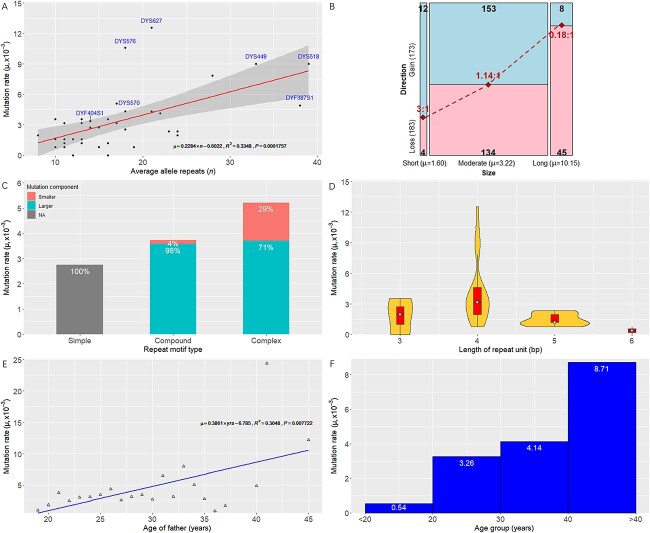
Sequence-based mutation patterns at Y chromosomal short tandem repeat (Y-STRs). (A) Positive linear relationship between the mutation rate and the average allele repeat number (slope = 0.2284; *R*^2^ = 0.3348; *P* = 1.76 × 10^−4^). The initially defined rapidly mutating Y-STR markers (DYF387S1a/b, DYF404S1a/b, DYS449, DYS518, DYS570, DYS576, and DYS627) are annotated. (B) Correlation between the allele size (short, moderate, and long) and the direction of mutation (gain and loss). The average mutation rate of long alleles is significantly greater than that of moderate and short alleles (*P* = 2.35 × 10^−12^). Longer alleles present a tendency towards repeat losses whilst shorter alleles towards repeat gains. (C) Average mutation rates of different repeat motif types (simple, compound, and complex). Significant differences are observed between simple–compound (*P* = 0.0201), simple–complex (*P* = 2.35 × 10^−12^), and compound–complex (*P* = 0.0250) as well as the component of larger *versus* smaller units occurring mutations between compound–complex (*P* = 2.20 × 10^−16^). (D) Average mutation rates of different repeat unit lengths. A small but negative linear is observed when the repeat unit ≥4 bp (slope = −2.1243, *R*^2^ = 0.1603, *P* = 0.0190). (E) Positive linear relationship between the mutation rate and the age of father at the gametogenesis (slope = 0.3861; *R*^2^ = 0.3048; *P* = 0.0077). (F) Average mutation rates of different father’s age intervals. The number of mutations is significantly increased with the age intervals (*P* = 0.0048).

Firstly, Figure 2A showed that the statistically significant linear relationship (*P* = 1.76 × 10^−4^) was observed between the number of average allele repeats and the mutation rate (Supplementary Table S6), i.e. the mutation rate was increased with the allele repeat number (slope = 0.2284), where 33.48% of the variance in the mutation rate might be explained by the allele repeat number and two outliers with remarkably high mutation rates were observed, that is, RM Y-STRs DYS576 and DYS627 (Section *Mutation rates*). If nine initially defined RM Y-STR loci (DYF387S1a/b, DYF404S1a/b, DYS449, DYS518, DYS570, DYS576, and DYS627) were removed, 70.14% of the variance in the mutation rate would be explained by the allele repeat number. According to Ge et al. [46], the allele repeat number based on allele frequencies in Supplementary Table S2 were classified into 25%, 50%, and 25% categories for short, moderate, and long allele sizes, respectively. Supplementary Table S7 showed the mutation rate of long-size alleles (10.1 × 10^−3^) is significantly (*P* = 2.35 × 10^−12^, Fisher’s exact test) greater than moderate-size (3.2 × 10^−3^) and short-size (1.6 × 10^−3^) alleles, which demonstrated mutations at longer alleles were more common than those at shorter ones and further validated the above-mentioned relationship between the allele repeat number and the mutation rate. Also, Figure 2B displayed the relationship between the allele size and the direction of mutation. The ratio of repeat gains *versus* losses in short, moderate, and long allele sizes demonstrated that longer alleles had a tendency towards repeat losses whilst shorter alleles towards repeat gains.

Secondly, Y-STR markers can be divided into simple, compound, and complex repeats based on their repeat motifs (Supplementary Table S6). Generally speaking, simple repeats contain repeat units of identical length and sequence, compound repeats comprise two or more adjacent simple repeats with commonly a difference of one nucleotide, and complex repeats embody multiple repeats with variable unit lengths and/or intervening sequences [47, 48]. Statistically significant differences in mutation rates were observed between simple and compound repeats (χ^2^ = 5.40, *P* = 0.0201), between compound and complex repeats (χ^2^ = 5.02, *P* = 0.0250), and between simple and complex repeats (χ^2^ = 24.77, *P* = 2.35 × 10^−12^), with complex repeats expressing a higher average mutation rate (5.2 × 10^−3^) than compound ones (3.7 × 10^−3^) and simple ones (2.7 × 10^−3^). Moreover, Figure 2C showed that the location of repeat units caused the mutational events in compound and complex repeats. A majority (95.79%) of mutational events occurred at the larger variable repeat units in compound repeats, e.g. DYS390: [TAGA]4 CAGA [TAGA]**10** [CAGA]8 → [TAGA]4 CAGA [TAGA]**11** [CAGA]8, whereas 70.97% of mutational events resulted from the larger variable units and 29.03% from smaller variable ones in complex repeats, e.g. DYF387S1a/b: [AAAG]3 GTAG [GAAG]4 [AAAG]2 GAAG [AAAG]2 [GAAG]**11** [AAAG]15 → [AAAG]3 GTAG [GAAG]4 [AAAG]2 GAAG [AAAG]2 [GAAG]**10** [AAAG]15. Extremely rare mutations were observed occurring at both larger and smaller variable repeat units in complex repeats, e.g. DYF387S1a/b: [AAAG]3 GTAG [GAAG]4 [AAAG]2 GAAG [AAAG]2 [GAAG]**10** [AAAG]**17** → [AAAG]3 GTAG [GAAG]4 [AAAG]2 GAAG [AAAG]2 [GAAG]**11** [AAAG]**15**. A statistically significant difference in the component of larger *versus* smaller units occurring mutations was observed between compound and complex repeats (χ^2^ = 86.78, *P* = 2.20 × 10^−16^).

Thirdly, the average mutation rate was 1.8 × 10^−3^ for the three trinucleotide Y-STRs, 4.1 × 10^−3^ for the 31 tetranucleotide Y-STRs, 1.5 × 10^−3^ for the five pentanucleotide Y-STRs and 0.4 × 10^−3^ for the two hexanucleotide Y-STRs. Figure 2D showed no obvious linear correlation was found between length of repeat unit and the mutation rate (*P* = 0.1426), but the differences in average mutation rates amongst the length of repeat units proved to be statistically significant (χ^2^ = 43.02, *P* = 2.43 × 10^−9^). Unlike the results of Ballantyne et al. [34] but like those of Claerhout et al. [47], the sequence-based mutation rates did not conform to the convention that the shorter repeat units the STR markers had, the higher the mutation rate went. In our study and Claerhout et al. [47], the average mutation rate for trinucleotide Y-STRs was significantly lower than that for tetranucleotide ones (χ^2^ = 8.45, *P* = 0.0037). If we remove those trinucleotide Y-STRs, there would be a small but statistically significant linear decrease in the mutation rate as the repeat unit length increased (slope = −2.1243, *R*^2^ = 0.1603, *P* = 0.0190).

Lastly, the average age of father at the gametogenesis was calculated as 26.29 ± 4.21 years and the median was 25 years, which was concordant with a generation time (25 years) usually assumed to estimate the time to the most recent common ancestor in evolutionary studies [47, 49]. Compared ages of fathers between with mutations (26.75 ± 4.10 years) and without mutations (26.23 ± 4.12 years), the statistically significant difference was observed (*P* = 0.0383, Welch Two Sample *t*-test). Figure 2E showed a statistically significant positive linear correlation (slope = 0.3861) between the mutation rate and the age of the father (*R*^2^ = 0.3048, *P* = 0.0077), which were also observed by Ballantyne et al. [34] and Claerhout et al. [47]. Further, ages were artificially demarcated by the 10-year interval for a male generation time interval most frequently ranges between 20 and 30 years [50]. The number of mutations was significantly increased with the age intervals (χ^2^ = 12.93, *P* = 0.0048), ranging from 0.5 × 10^−3^ of the average mutation rate within <20-year interval to 8.7 × 10^−3^ within >40-year interval (Figure 2F).

### StatsY

StatsY v1.0 runs on Windows® 7–10 operating systems with the 64-bit processor architecture and offers Chinese and English display languages. The code is mainly divided into three modules: “quality control” module that is designed for screening the format of an input file and data included and providing QC information; “haplotype” module for searching shared haplotypes and calculating allele/haplotype frequencies and forensic parameters; and “mutation” module for counting mutational events, directions, and steps at each locus by each father–son pair, estimating mutation rates and their binomial 95%CIs in a dataset and displaying the bar graph. All calculation methods and formulae were provided on Section *Statistical analysis*.

StatsY v1.0 only accepts a Microsoft Excel workbook (^*^.xlsx) as the input file. In “haplotype” module, the first column mandatorily provides the user-defined sample identifier (e.g. NHC0001) to trace a sample with missing data and the first row mandatorily provides the locus name (e.g. DYS19) in a workbook. In “mutation” module, the first column must provide the same pedigree identifier by each two rows (e.g. one P00001 for father and the other P00001 for son); the second column must designate “P” for father and “O” for son; the first row must provide the locus name as well. The number of loci to be calculated is unlimited. Allele data are entered with one column per locus but alleles at multi-copy markers are treated as genotypes and separated by a comma (e.g. DYS385a/b: 13,13 for a homozygote and 13,20 for a heterozygote). Allele repeats must be positive and intermediate alleles can include one decimal place (e.g. 23.2). Missing data must be left with an empty cell. An allele with non-positive number and text (e.g. −1 or 0; NA or 23.x) or missing data will be automatically ignored in the calculations. For convenience, two templates of the input files (“Template for Haplotype.xlsx” and “Template for Mutation.xlsx”, respectively) are provided under the installation directory.

**Figure 3:**
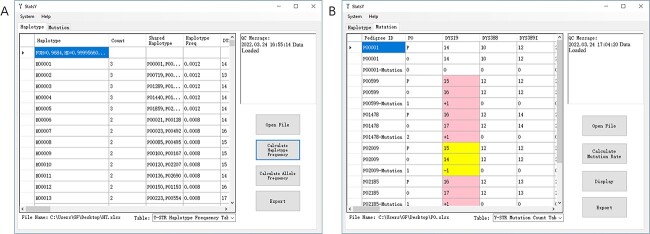
User interface of StatsY. (A) “Haplotype” sheet and (B) “Mutation” sheet. Details were explained in Section *StatsY.*

The user interface of StatsY v1.0 was shown in Figure 3. After importing an input file for haplotypes or mutations, StatsY v1.0 returned a data table on the left panel that could be investigated and exported as an EXCEL workbook and also prompted QC messages on the right panel to indicate samples or pedigrees including invalid or missing data that would be automatically ignored in the proceeding calculations. In “Haplotype” sheet, allele frequencies for all loci and for multi-copy loci included in the input file, haplotype frequencies, and their forensic parameters could be checked on the left panel and exported as EXCEL workbooks after clicking on “Calculate Allele Frequency” and “Calculate Haplotype Frequency” buttons on the right panel. In “Mutation” sheet, mutation counts (events, directions, and steps) and mutation rates could be also checked and exported after clicking on “Calculate Mutation Rate” button. Additionally, allele frequencies based on father samples in father–son pairs and mutation events and directions (pink colour for gain and purple colour for loss) directly over certain alleles could be plotted by locus to inspect hotspot distributions and saved as a Bitmap (BMP) file to archive once “Display” button was clicked on the right panel. Documentation for StatsY v1.0 is provided under the installation directory.

## Conclusions

In this article, we present haplotypes and estimate mutation rates at 41 Y-STRs in 2 548 father–son pairs from a Northern Han Chinese population. Allele frequencies range from 0.0002 to 0.9447 and the average GD value is 0.6934 with the range from 0.1051 to 0.9657. We detect 2 387 unique haplotypes in 2 548 father samples with the HD and DC values of 0.999 956 608 and 0.96 741 007. Sequencing analysis of 24 overlapped Y-STRs between the MPS Kit and the CE Kit from 308 father samples in mutational pairs reveals that 39.53% more polymorphisms from sequence-based alleles is obtained than that from length-based alleles and the average GD value increases as well. Moreover, the average mutation rate at 41 Y-STRs is ⁓3.4 × 10^−3^ (Binomial 95%CIs: 3.1 × 10^−3^–3.8 × 10^−3^), which is comparable to other studies. The mutation rates at DYS576 and DYS627 are higher than 1 × 10^−2^ in Northern Han Chinese, whilst the mutation rates at DYF387S1a/b, DYF404S1a/b, DYS449, DYS518, and DYS570 are lower than initially defined. Most mutational events occur at a single locus and result in one-step differences. No bias for repeat gains *versus* losses is observed. The molecular factors known to drive Y-STR mutation rates increased such as the longer STR region, the more complex motif, and the order father have been all confirmed in this study, but the sequence-based mutation rates do not conform to the convention that Y-STR with a shorter repeat unit will have a higher mutation rate. Further, StatsY is developed to be the interactive graphical and installable software for forensic scientists, which can facilitate to calculate allele and haplotype frequencies, forensic parameters, and mutation rates at Y-STRs.

## Supplementary Material

Supplemental_Materials_Tables_S1-S7_owad016
